# Nature of Charge Carrier Recombination in CuWO_4_ Photoanodes for Photoelectrochemical Water Splitting

**DOI:** 10.1021/acsaem.3c01608

**Published:** 2023-09-20

**Authors:** Ivan Grigioni, Annalisa Polo, Chiara Nomellini, Laura Vigni, Alessandro Poma, Maria Vittoria Dozzi, Elena Selli

**Affiliations:** Dipartimento di Chimica, Università degli Studi di Milano, Via Golgi 19, 20133 Milano, Italy

**Keywords:** photoelectrocatalysis, ternary oxides, solar water splitting, solar light, renewable energy

## Abstract

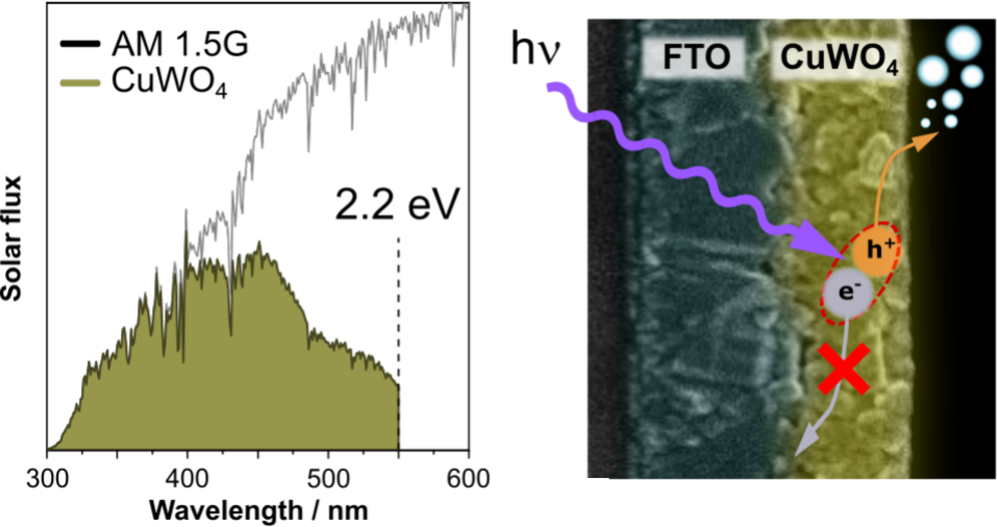

CuWO_4_ is a ternary semiconductor oxide with excellent visible light harvesting properties up to 550 nm and stability at high pH values, which make it a suitable material to build photoanodes for solar light conversion to hydrogen via water splitting. In this work, we studied the photoelectrochemical (PEC) performance of transparent CuWO_4_ electrodes with tunable light absorption and thickness, aiming at identifying the intrinsic bottlenecks of photogenerated charge carriers in this semiconductor. We found that electrodes with optimal CuWO_4_ thickness exhibit visible light activity due to the absorption of long-wavelength photons and a balanced electron and hole extraction from the oxide. The PEC performance of CuWO_4_ is light-intensity-dependent, with charge recombination increasing with light intensity and most photogenerated charge carriers recombining in bulk sites, as demonstrated by PEC tests performed in the presence of sacrificial agents or cocatalysts. The best-performing 580 nm thick CuWO_4_ electrode delivers a photocurrent of 0.37 mA cm^–2^ at 1.23 V_SHE_, with a 7% absorbed photon to current efficiency over the CuWO_4_ absorption spectrum.

## 1 Introduction

Photoelectrochemical (PEC) water splitting produces clean O_2_ and H_2_ from water by exploiting sunlight directly.^1^ The oxygen evolution reaction requires anodic semiconductor materials that are resistant to harsh reaction conditions. Moreover, an ideal material needs a narrow band gap to absorb a considerable portion of visible light and guarantee a solar to hydrogen efficiency above 10%, which is the lower limit for industrialization.^2,3^

Binary metal oxide-based photoelectrodes, such as WO_3_ and α-Fe_2_O_3,_^4,5^ initially attracted interest as visible-light-active materials. WO_3_ has a high electron diffusion length and mobility,^4,6−8^ and optimized electrodes allow almost complete absorbed photons to current conversion efficiency. However, WO_3_ has poor chemical stability in neutral or basic pH and a relatively wide band gap (2.6–2.7 eV). On the other hand, hematite (Fe_2_O_3_) has an intriguing narrow band gap of 2.0 eV with potential solar to hydrogen conversion efficiency (η_SHE_) up to 15% (Table 1),^9^ but limited charge carrier lifetime (below 1 ns in undoped Fe_2_O_3_)^10,11^ and mobility, which translate into suboptimal PEC performances.

**Table 1:** Commonly Used Semiconductor Oxide Photoanodes

semiconductor	BG^a^/eV	*J*_max_^b^/mA cm^–2^	η_SHE_^c^
WO_3_	2.6	4	5%
BiVO_4_	2.4	7	8%
CuWO_4_	2.2	10	12.5%
Fe_2_O_3_	2.0	12.5	15%
CuMoWO_4_	2.0	12.5	15%

a bang gap (BG).

b maximum current density (*J*_max_).

c solar to hydrogen efficiency (η_SHE_) of semiconductor photoanodes assembled in a two-electrode tandem PEC cell, assuming complete light absorption and conversion of photons with energy larger than the band gap and 100% faradaic efficiency to H_2_ and O_2._

More recently, ternary oxides such as BiVO_4_ with an intermediate band gap of 2.4 eV emerged as highly efficient materials for photoelectrocatalytic applications.^12−16^ State-of-the-art BiVO_4_ photoanodes show close-to-unity incident photon to current efficiency up to 480 nm.^17^ However, the band gap of BiVO_4_ is slightly larger than that desired for optimal photoanode materials. Indeed, a charge carrier recombination-free BiVO_4_ electrode (i.e., with an incident photon to current efficiency close to 100% up to the absorption onset at 520 nm) would lead to only 7–8% solar energy to hydrogen conversion, which implies that BiVO_4_ should be used in combination with narrower band gap materials in complex multilayer photoanodes.^18^

Ternary CuWO_4_ oxide has a band gap of 2.2 eV, and it could achieve an η_SHE_ of 12.5%.^19^ Furthermore, the CuWO_4_ band gap can be narrowed by substituting tungsten with molybdenum in quaternary CuW_*x*_Mo_*y*_O_4_. For example, CuW_0.5_Mo_0.5_O_4_ has a band gap of 2.0 eV, corresponding to an absorption edge of 650 nm.^20−22^ This tungstate family is stable in neutral and slightly basic pH because of the overlap between Cu 3d orbitals with O 2p orbitals.^23,24^ This hybridization is also responsible for the negative shift of the CuWO_4_ valence band compared to that of binary WO_3_.^25^ Moreover, CuWO_4_ is suitable to assemble heterojunction systems with BiVO_4_,^26,27^ likewise WO_3_.^28−32^

In prior studies, we investigated the charge carrier dynamics in transparent CuWO_4_ electrodes and found that charge carriers mostly recombine within a 10 ps time scale, with only ca. 10% of them surviving longer than 1 ns to potentially run water oxidation.^33^ In this work, we sought to experimentally study the intrinsic limits that hamper CuWO_4_ photoactivity by employing a suite of PEC experiments to understand the factors shaping its performance. We prepared a series of thin-film CuWO_4_ electrodes through a citrate-based aqueous solution method and tuned the CuWO_4_ thickness to increase visible light exploitation of the photoanodes. To seek information on the charge carrier recombination processes occurring in bulk and surface CuWO_4_ and on their implication in the overall PEC performance, we tested the electrodes under front- and back-side irradiation in both simulated solar light and monochromatic irradiation experiments. We also varied the light intensity to study the effect of the charge carrier density on the PEC activity and found that the PEC performance of CuWO_4_ is light-intensity-dependent, with charge recombination increasing with light intensity, and that most photoproduced charge carriers recombine in bulk sites.

## 2 Experimental Section

### 2.1 Materials

The following chemicals were employed in the present work: copper(II) nitrate trihydrate (99% purity, Sigma-Aldrich), ammonium metatungstate hydrate (NH_4_)_6_H_2_W_12_O_40_·*x*H_2_O (99% purity, Fluka), citric acid (99% purity, Sigma-Aldrich), deionized water, boric acid (99.5% purity, Sigma-Aldrich), and KOH (99.5% purity, Sigma-Aldrich). All chemicals were used as received, with no further purification.

### 2.2 Photoelectrodes Preparation

The CuWO_4_ thin films were prepared following a previously reported procedure.^33^ Specifically, a 0.5 M solution of CuWO_4_ was prepared by dissolving 14 mmol of citric acid in 5.3 mL of ethanol and 2.4 mL of deionized H_2_O, followed by the addition of 5 mmol of Cu(NO_3_)_2_·3H_2_O under vigorous stirring and a stoichiometric amount of ammonium metatungstate hydrate. Fluorine-doped tin oxide (FTO) glass (Pilkington Glass, TEC-7, thickness of 2 mm) was used as conductive glass to prepare the FTO/CuWO_4_ electrodes. The FTO glass was cleaned under sonication for 30 min in a soap-water solution, rinsed thoroughly with water, sonicated for 30 min in ethanol, and then dried in air. After cleaning, the electrodes were prepared by spin coating 100 μL of the CuWO_4_ solution on FTO, at 4000 rpm for 30 s. Then, the CuWO_4_ film was preannealed at 250 °C for 10 min and then annealed at 550 °C for 1 h. Prior to any test, the photoanodes were treated for about 30 s in a 0.5 M HCl aqueous solution to eventually eliminate any trace of CuO, then washed with distilled water and dried in air. The deposition, annealing, and HCl cleaning steps were repeated up to six times to prepare multilayer electrodes with the desired thickness and light absorption properties.

The NiFeO_*x*_ cocatalyst was deposited onto the photoelectrodes employing a hydrothermal method,^34^ by soaking the CuWO_4_ electrode into an aqueous solution containing NiCl_2_ (0.2 mM) and FeCl_3_ (29.8 mM) as Ni^2+^ and Fe^3+^ sources in a closed vessel for 45 min at 100 °C.

### 2.3 Optical, Morphological, Structural, and Photoelectrochemical Tests

The absorption spectra of the CuWO_4_ thin films on the electrodes were recorded in transmission mode with a Jasco V-650 spectrophotometer. The crystalline phase of the photoactive materials was determined by XRD analysis of the deposited thin films, using a Philips PW 1830/40 X-ray powder diffractometer equipped with a Cu tube at 40 kV and 40 mA. Top-view and cross-sectional field emission scanning electron microscopy (FESEM) images were acquired employing a LEO 1430 scanning electron microscope operating at a 10 kV accelerating voltage and an 8 mm working distance.

PEC measurements were performed using a homemade cell and an Autolab PGSTAT 12 potentiostat controlled by the NOVA software. In a typical setup, the electrode was used as the working electrode, a Pt wire was used as the counter electrode, and Ag/AgCl (3.0 M in NaCl) was used as the reference electrode. The photoanodes were tested under both back-side irradiation (through the FTO/CuWO_4_ interface) and front-side irradiation (through the CuWO_4_/FTO interface). The light source was an Oriel, Model 81172 Solar Simulator equipped with an AM 1.5 G filter. In simulated solar light irradiation experiments, the light intensity was measured with a Thorlab PM200 power meter equipped with a Si power head (S130VC) and set at 100 mW cm^–2^ (1 sun). In PEC experiments under simulated solar light with different intensities, a neutral light attenuator filter was employed to obtain 25 and 50 mW cm^–2^ intensities (0.25 and 0.5 sun, respectively) and a quartz lens was used to increase the intensity to 150 and 200 mA cm^–2^ (1.5 and 2 suns, respectively).

PEC experiments were carried out in a 0.1 M potassium borate (KBi) buffer solution at pH 9 unless otherwise stated. The potential vs Ag/AgCl was converted into the standard hydrogen electrode (SHE) scale using the following equation
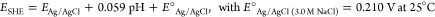


The incident photon-to-current efficiency (IPCE) was measured using a 300 W Lot-Oriel Xe lamp equipped with a Lot-Oriel Omni-λ 150 monochromator and a Thorlabs SC10 automatic shutter. A 1.23 V bias vs SHE (V_SHE_) was applied, and the current was measured with a 10 nm step within the 350 to 600 nm wavelength range. The incident light power was measured at each wavelength by using a calibrated Thorlabs S130VC photodiode connected to a Thorlabs PM200 power meter. The IPCE at each wavelength was calculated using the following equation
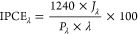
where *J*_λ_ is the photocurrent density (mA cm^–2^) and *P*_λ_ (mW cm^–2^) is the power of the monochromatic light at wavelength λ (nm).

The internal quantum efficiency (IQE) at each wavelength was calculated by combining the IPCE curve with the absorption (*A*) spectrum of each photoanode
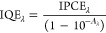


## 3 Results

### 3.1 Characterization of CuWO_4_ Multilayer Photoanodes

**Figure 1:**
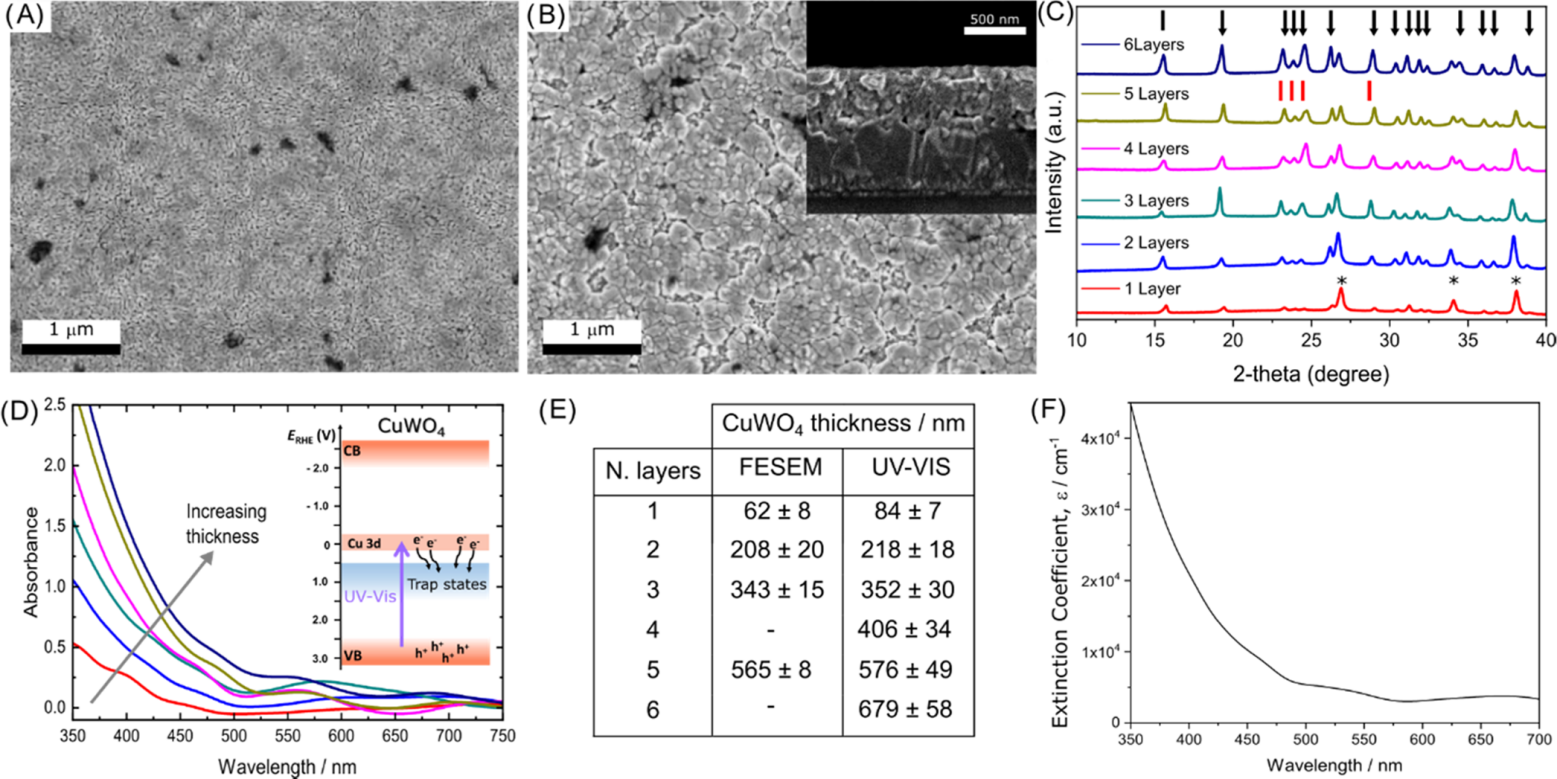
Field emission scanning electron microscopy (FESEM) top views of (A) 2 layers and (B) 5 layers of CuWO_4_. Inset in (B): cross section of the CuWO_4_ film obtained by depositing 5 layers. (C) XRD patterns of the CuWO_4_ electrodes. The diffraction signals of WO_3_ (JCPDF 89–4476), CuWO_4_, and underlying FTO are identified with red bars, black arrows, and asterisks, respectively. (D) Absorption spectra of the CuWO_4_ photoanodes with increasing semiconductor thickness. Inset: scheme of the optical transitions in CuWO_4_. (E) Thickness of the electrodes with 1–6 CuWO_4_ layers, from cross-sectional FESEM images (left column) and calculated from the absorption coefficient at 420 nm (right column). (F) Extinction coefficient vs wavelength of a 5-layered CuWO_4_ electrode.

We started by studying the morphology of the CuWO_4_ photoanodes by field emission scanning electron microscopy (FESEM). Top-view images (Figure 1A,B) reveal that CuWO_4_ progressively covers the FTO substrate uniformly. Two deposited layers provide a continuous coating film consisting of oval-shaped nanostructures (Figure S1 in the Supporting Information). Bigger grains form in thicker electrodes (Figure 1B).

Cross-sectional images (inset of Figures 1B and S2) show that the CuWO_4_ coating is compact along its whole thickness. Therefore, we could estimate the thickness of the photoactive layer in electrodes prepared with 1, 2, 3, and 5 CuWO_4_ deposition cycles (Figure 1E).

The phase purity of the CuWO_4_ films was assessed through XRD analysis and comparison with the Bragg reflections from JCPDF 72–0616, which revealed a triclinic structure (Figure 1C) and the absence of the WO_3_ phase in all electrodes. Although no CuO phase is detectable in the XRD patterns, we washed the electrodes with diluted HCl to dissolve possible CuO impurity traces.

Then, we studied the optical properties of the electrodes by absorption spectroscopy in transmittance mode (Figure 1D). The multilayer films are optically transparent, with the absorbance in the UV region steeply increasing below 450 nm, without obvious absorption peaks consistently with the indirect forbidden d–d transition occurring in this Mott–Hubbard semiconductor,^35−37^ possibly originating light scattering at wavelengths longer than 550 nm.

The main optical transition of CuWO_4_ is assigned to the photoexcitation of electrons from the valence band, composed of O 2p states partly mixed with Cu 3d states, to the conduction band—empty Cu 3d levels (inset of Figure 1D).^33^ A ca. 2.1 eV energy gap separates the band edges. CuWO_4_ has an additional band of empty states at higher energy, i.e., at about 5 eV above the top of the valence band, consisting of W 5d and Cu 3d states. At wavelengths above 550 nm, scattering appears in the absorption spectrum of thicker films, possibly originating from the bigger grains observed in Figure 1B.

We combined the thickness values obtained from cross-sectional FESEM images and the absorption spectrum of CuWO_4_ to estimate the absorption coefficient of CuWO_4_ films at 420 nm (the edge between UV and visible light), using the following equation
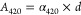
where α_420_ (cm^–1^) is the absorption coefficient of CuWO_4_ at 420 nm, *A*_420_ is the absorption at 420 nm, and *d* is the average thickness in cm calculated from the cross-sectional FESEM images. From the *A*_420_ vs film thickness plot (see Figure S3), we calculated the absorption coefficient for CuWO_4_ at 420 nm, α_420_ = (1.65 ± 0.14) 10^4^ cm^–1^. This value is rather low compared to other semiconductor oxides, consistent with the indirect nature of the CuWO_4_ band gap. For instance, it is 4-fold lower than the α_420_ value of BiVO_4_ (6.7 × 10^4^ cm^–1^),^38^ and this implies that to absorb the same amount of 420 nm photons, a CuWO_4_ electrode needs to be 4 times thicker than a BiVO_4_ electrode. Interestingly the absorption coefficient for CuWO_4_ at 420 nm is also ca. 2-fold lower compared to CuW_0.5_Mo_0.5_O_4_ (3.41 × 10^4^ cm^–1^),^20^ which supports the hypothesis that Mo doping introduces additional optical transitions in CuWO_4_.

The α_420_ for CuWO_4_ value was then used to evaluate the thickness of the prepared CuWO_4_ films from their absorbance at 420 nm. These thickness values are reported in Figure 1E. For convenience, hereafter, we identify the electrodes with different CuWO_4_ thicknesses as CuWO_4_:X, with X referring to the CuWO_4_ thickness in nanometers (rounded to the nearest ten).

We also calculated the extinction coefficient of CuWO_4_ at all wavelengths, based on the overall absorption spectrum of the thinnest electrode (which is moderately affected by scattering), as , where *A*_λ_ is the absorbance over the 300–800 nm interval and *d* is the film thickness. The so estimated extinction coefficient of CuWO_4_ vs wavelength is shown in Figure 1F.

### 3.2 Photoresponse of CuWO_4_ Multilayer Photoanodes

We performed PEC experiments under monochromatic and simulated solar light irradiation to probe the photoresponse of the CuWO_4_ electrodes with increasing thickness of the semiconductor layer. The experiments under simulated solar light provide information on their performance under conditions close to field application, while PEC tests performed under monochromatic irradiation provide information on the irradiation wavelength-dependent behavior of photoactive materials. We also tested the electrodes under back-side and front-side irradiation, i.e., with the light reaching CuWO_4_ through the FTO or the electrolyte, respectively. Indeed the comparison between the PEC performance in the two modes proved diagnostic for identifying electron or hole transport as a limiting process in the illuminated semiconductor.^39,40^

**Figure 2:**
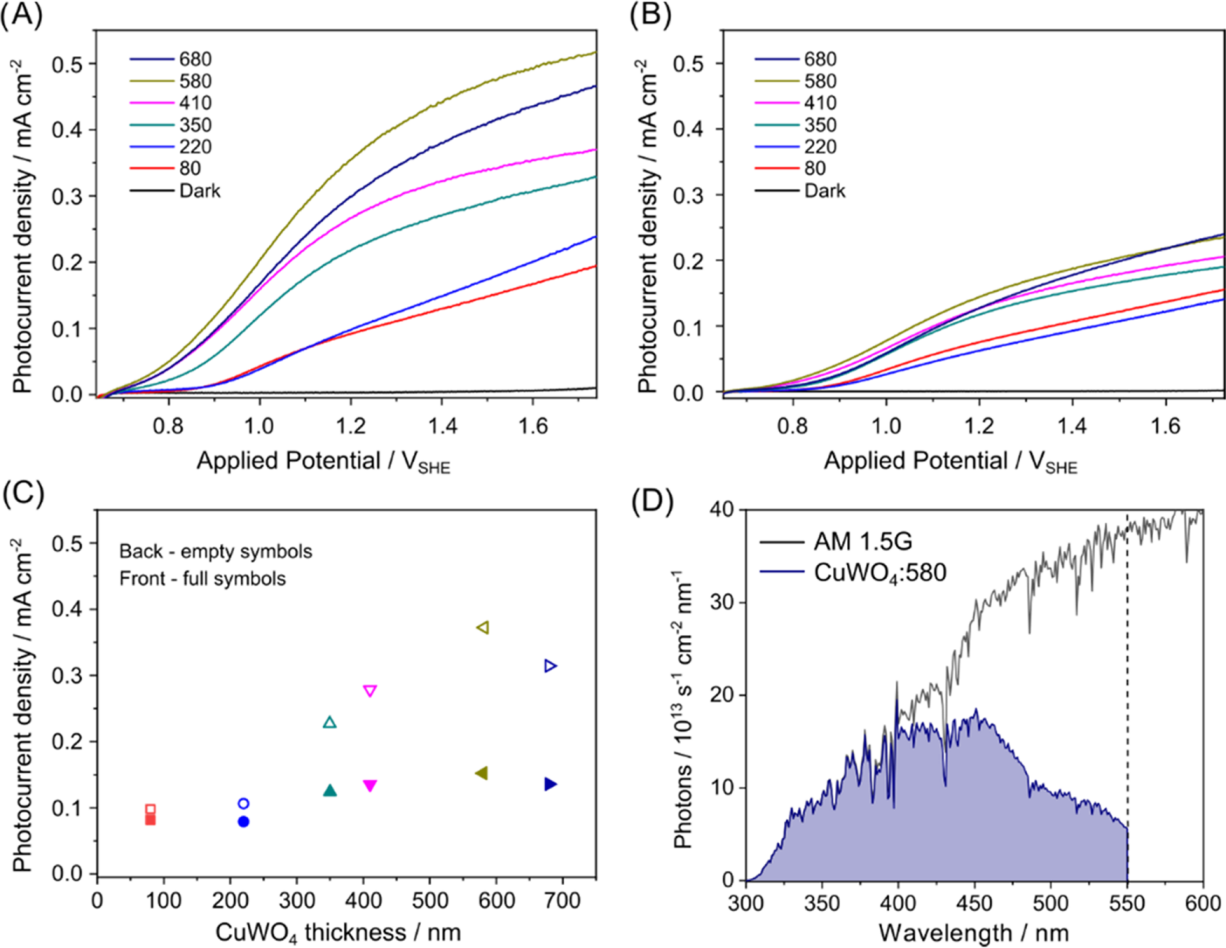
Linear sweep voltammetry (LSV) curves recorded with CuWO_4_ multilayer photoanodes under (A) back-side or (B) front-side simulated solar light irradiation (100 mW cm^–2^) in 0.1 M KBi buffer solution at pH 9. The labels within the figures refer to the thickness of the CuWO_4_ layers, in nm. (C) Current density at 1.23 V_SHE_ as a function of the CuWO_4_ thickness, under back- and front-side irradiation (empty and full symbols, respectively). (D) Overall photon flux in the AM 1.5 solar spectrum (black trace) and number of photons absorbed per second by CuWO_4_:580 (blue area). The dashed vertical line marks the CuWO_4_ absorption edge.

Linear sweep voltammetry (LSV) tests performed under simulated solar light irradiation in 0.1 M borate buffer solution (KBi buffer) evidence that the photocurrent vs voltage increases with the CuWO_4_ layer thickness (Figure 2A,B), up to a 580 nm thick CuWO_4_ photoactive layer. Under back-side irradiation, the electrodes generate a larger photocurrent than under front-side irradiation (Figure 2C). For instance, the photocurrent of the CuWO_4_:580 electrode under back irradiation is ≥2x than in the front-side mode.

Indeed, under front-side irradiation, most electrons are photogenerated far from the electron extraction site (the contact with the FTO layer). The lower photocurrent, compared to back-side irradiation, could indicate that electrons in CuWO_4_ can travel only short distances because they undergo recombination with photogenerated holes within the film thickness, before reaching the extraction site. With the thinnest CuWO_4_:80 electrode, similar photocurrents are recorded under front- and back-side irradiation (Figure 2A,B), demonstrating that photopromoted electrons can diffuse efficiently at least within a ca. 80 nm distance.

The best-performing CuWO_4_:580 electrode generates a photocurrent density of 0.37 mA cm^–2^ at 1.23 V_SHE_ under back-side irradiation. This value is in line with previous records for CuWO_4_-based electrodes prepared with different synthetic strategies (Table 2).^41,42^ However, it is far from the maximum photocurrent expected for the 2.2 eV band gap of CuWO_4_. Indeed, assuming that all absorbed photons in the CuWO_4_:580 electrode (Figure 2D) are completely converted into current would lead to a 5.6 mA cm^–2^ theoretical maximum photoresponse under 1 sun illumination. Thus, the photogenerated current is only 6.7% of this maximum value, which implies that more than 90% of the photogenerated charge carriers undergo recombination.

**Table 2:** Photoelectrochemical Performances of Literature Benchmark CuWO_4_ Photoanodes Prepared with Different Synthetic Approaches

			*J*^g^	stability		
synthetic method	electrolyte	thickness/nm	/ mA cm^–2^	Time / min	J_fin_^*h*^ / mA cm^-2^^–2^*j*_fin_^h^	ref
spin-coating	0.1 M KPi (pH 7)	580	0.37	120	0.37	this work
one step-hy^a^	0.2 M KPi (pH 7)^e^	∼1500	0.38	50	0.3	(43)
ST – hy^b^	0.1 M KPi (pH 7)	≥1000	∼0.3	60	∼5%^i^	(44)
ST – hy	0.1 M KPi (pH 7)	≥1000	0.34	240	∼35%^i^	(45)
ST – hy	1 M KBi (pH 9)^f^	≥1000	0.4	60	0.39	(29)
ST – hy	0.1 M KPi (pH 7)	2000	0.42	n/a	n/a	(46)
ST – hy	0.1 M KPi (pH 7)	≥1000	0.35	240	0.26	(47)
spin-coating	KPi (pH 7)	500	0.47	600	0.4	(48)
e-deposition^c^	0.1 M KPi (pH 7)	2–3000	0.18	600	∼50%^i^	(25)
ALD^d^	1 M KBi (pH 9.0)	80	∼0.15	n/a	n/a	(41)
ST - hy	0.1 M Kpi (pH 7)	≥1000	0.35	60	∼17%^i^	(22)
spin coating	0.1 M Na_2_SO_4_ (pH 6.8)	800	0.27	2.5	0.21	(49)
spray pyrolysis	0.1 M KPi (pH 7)	1.5–2000	0.19	n/a	n/a	(50)
spin coating	KPi/NaCl 0.1/0.14 M	250	0.38	n/a	n/a	(51)
ST – hy	0.1 M KPi (pH 7)	≥1000	0.38	240	0.39	(52)

a one-step hydrothermal.

b sacrificial template–hydrothermal.

c electrodeposition.

d atomic layer deposition.

e potassium phosphate buffer.

f potassium borate buffer.

g current density at 1.23 V_SHE_,

h current density at 1.23 V_SHE_ at the end of the stability test,

i current density percent drop at the end of the stability test (in this case the stability is at a voltage ≥1.23 V_SHE_).

**Figure 3:**
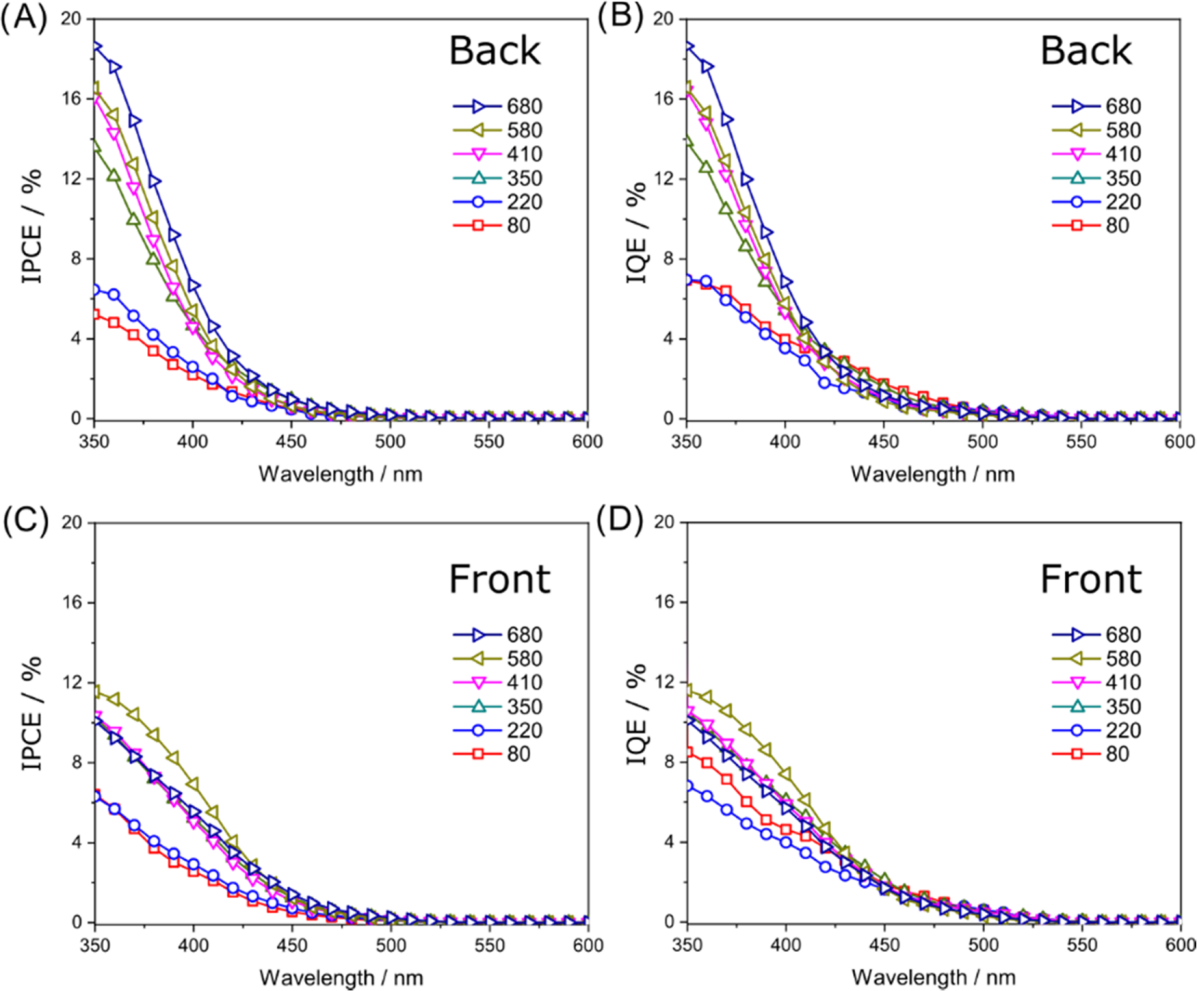
(A, C) IPCE and (B, D) IQE analyses of CuWO_4_ multilayer photoanodes under (A, B) back-side and (C, D) front-side monochromatic irradiation, at 1.23 V_SHE_.

Figure 3 shows the incident photon to current efficiency (IPCE) and internal quantum efficiency (IQE) curves recorded with the CuWO_4_ photoanodes under back- and front-side irradiation at an applied bias of 1.23 V_SHE_. All IPCE curves monotonously decrease with increasing incident wavelength without any defined peak or steep IPCE feature (Figure 3A,C). This suggests the presence of a single electronic transition with broad rather than sharp band edges within the investigated light energy. The IQE values (Figure 3B,D), which account for the number of absorbed photons which generate photocurrent and is equal to the charge transport efficiency,^53^ are larger than the IPCE values. However, in this photoelectrode series, the IQE differs slightly from the corresponding IPCE values because of the relatively high electrode absorbance, especially in the UV region where the electrodes completely absorb the incident light. On the other hand, under visible light irradiation, when the electrodes absorb less than 50% of the incident light, the IQE is noticeably larger than the IPCE (Figure S4). The tests performed under monochromatic irradiation with the thickest electrodes pinpoint a CuWO_4_ photocurrent onset at ca. 550–560 nm (we measured IPCE at high applied bias, 1.73 V_SHE_, to increase the photocurrent signal close to the band gap excitation edge; see Figures S5 and S6), in good agreement with the reported 2.2–2.3 eV band gap of CuWO_4_.

Under back-side irradiation, the CuWO_4_ electrodes generate larger IPCEs than under front-side irradiation, implying that electron transport issues limit the overall photoresponse of this material and substantiating the behavior observed in LSV tests.^39^

Notably, under back-side irradiation, in LSV tests CuWO_4_:580 outperforms the other electrodes (Figure 2A), while in IPCE, the thickest CuWO_4_:680 electrode is best performing (Figure 3A). This difference relies on both the different light intensities (100 mW cm^–2^ in LSV analyses vs a few mW cm^–2^ in IPCE) and the poor electron transport of CuWO_4_. Under back-side irradiation and the low light intensity employed in IPCE analyses, most photons are absorbed close to the FTO glass where electron extraction toward the external circuit easily occurs. However, a larger light intensity (e.g., under simulated solar light irradiation) generates a larger amount of excited electrons deep in the CuWO_4_ film, far from FTO, at distances exceeding the mean electron diffusion within CuWO_4_. These electrons are more likely to recombine with holes and limit the overall photocurrent in the thickest electrode.

### 3.3 Light Intensity Dependence

**Figure 4:**
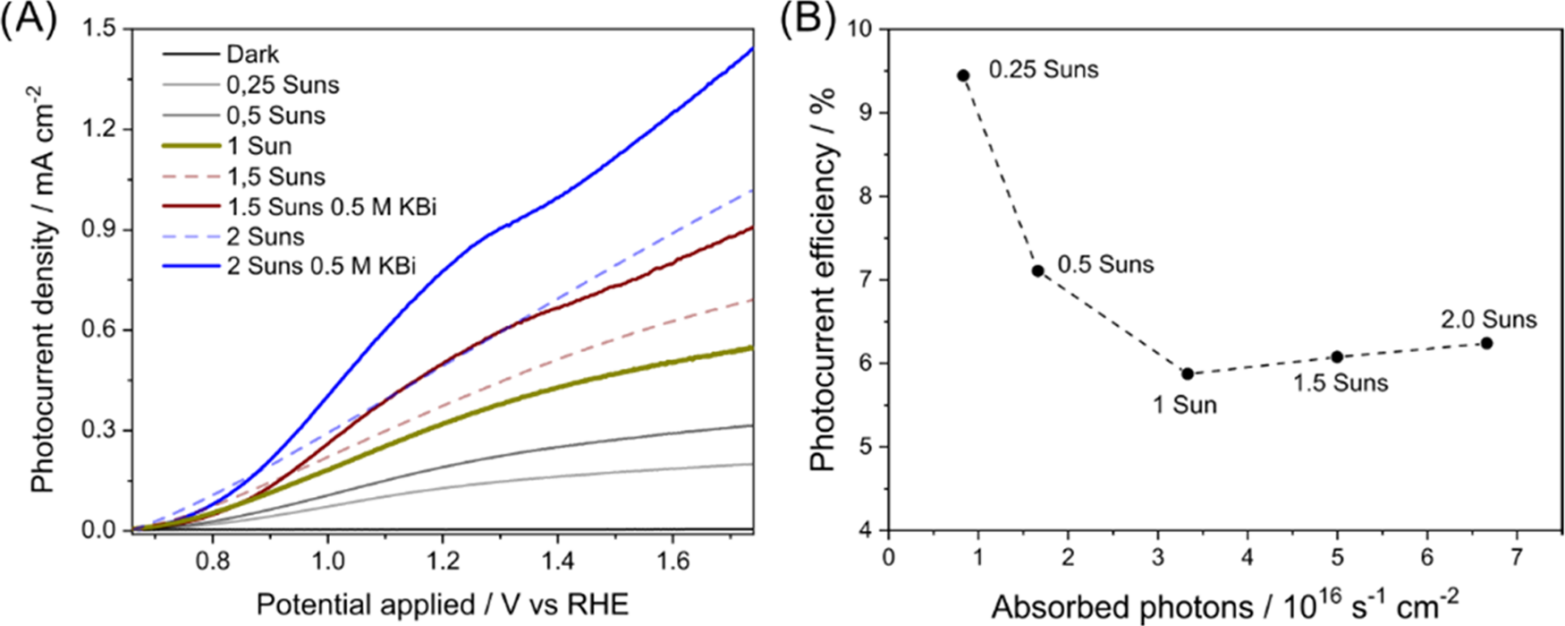
(A) LSV curves recorded with the CuWO_4_:580 photoelectrode under different irradiation intensities in contact with 0.1 M (0–1 sun) or 0.5 M (1.5–2 sun) (continuous lines) KBi solutions. The two dashed lines are the LSV curves recorded under 1.5 and 2 sun irradiation in contact with 0.1 M KBi solutions. (B) Photocurrent efficiency at 1.23 V_SHE_ vs absorbed photons per unit time at different irradiation intensities.

Employing the best-performing CuWO_4_:580 photoelectrode, we evaluated the effect on photoactivity of the light intensity under back-side irradiation, to get further information on the charge separation efficiency within the CuWO_4_ electrodes. For intensities larger than 1 sun, the measurements were carried out with the electrode in contact with a 0.5 M KBi electrolyte solution because the 0.1 M KBi electrolyte employed in LSV analyses under 1 sun irradiation (Figure 2) was insufficiently conductive to sustain photocurrents larger than 0.5 mA cm^–2^ (see dashed lines in Figure 4A).

The photocurrent response of the CuWO_4_:580 photoelectrode increases with increasing light intensity (Figure 4A), though this increase is not linear. We thus calculated the absorbed photons to current efficiency (photocurrent efficiency) over the full solar spectrum at different light intensities by dividing the number of photopromoted electrons exiting the CuWO_4_ film at 1.23 V_SHE_ by the total number of absorbed photons (Figure 2D, integrated blue area of the absorbed photons per nm). Figure 4B reports the photocurrent efficiency values vs the amount of absorbed photons per unit time.

The photocurrent efficiency decreases with increasing light intensity from 0.25 to 1 sun and stabilizes at ca. 6% for an illumination intensity greater than 1 sun (∼3 × 10^16^ s^–1^ cm^–2^). The drop from 0.25 to 1 sun is consistent with an increased charge carrier recombination at high photon flux, as found when comparing IPCE and LSV results, and could be related to inefficient bulk charge extraction at high light intensity.

### 3.4 Water Oxidation Kinetics of CuWO_4_

**Figure 5:**
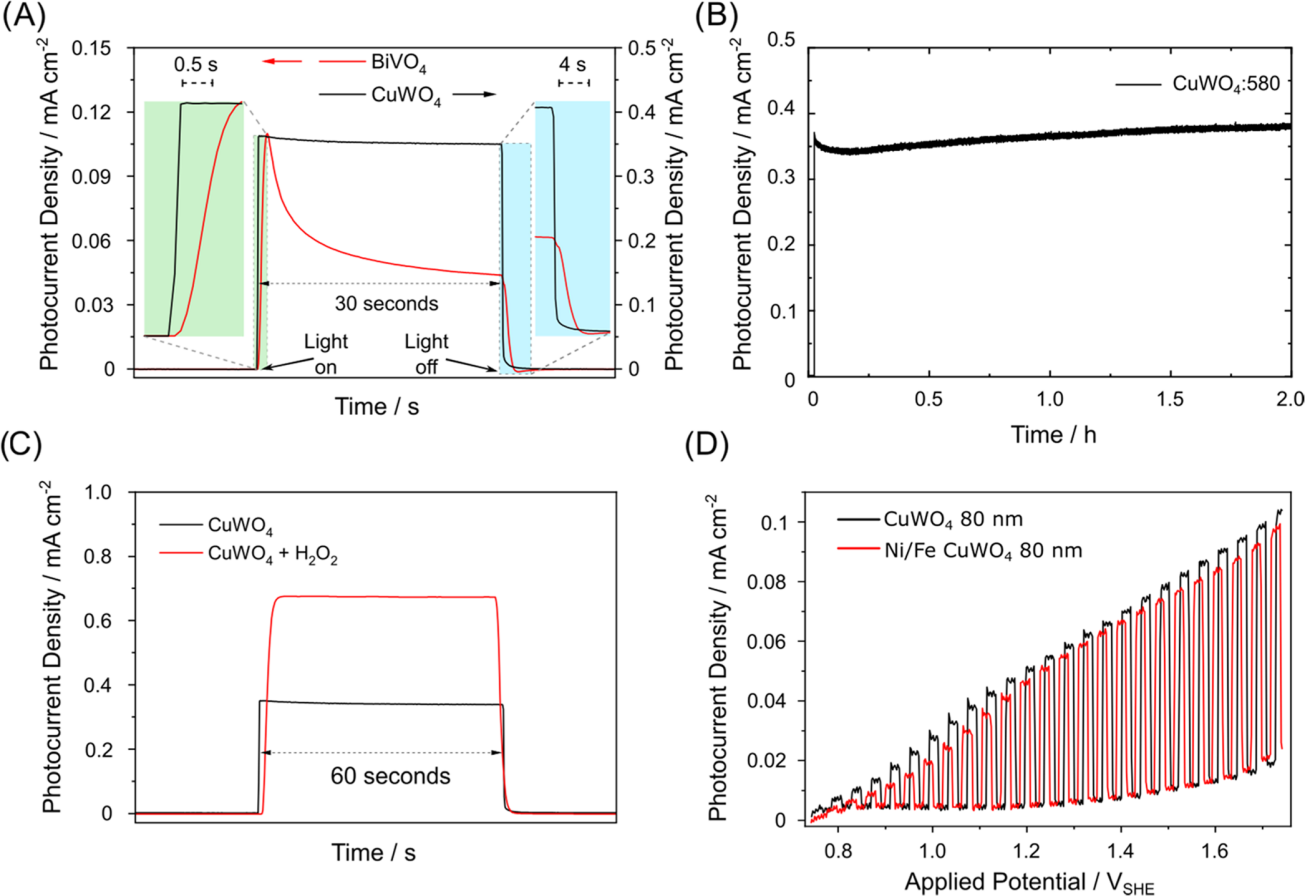
(A) Chopped chronoamperometry under 1 sun irradiation of an 80 nm thick CuWO_4_ photoanode (black line) and a 75 nm thick BiVO_4_ photoanode (red line), under back-side irradiation at 1.23 V_SHE_. The green (light on) and blue (light off) boxes display expanded portions of the photocurrent curve. (B) Chronoamperometric stability test of the CuWO_4_:580 electrode under simulated solar light irradiation in 0.1 M KBi. (C) Chopped chronoamperometry under 1 sun irradiation of the CuWO_4_:580 photoanode in the presence (red line) or in the absence (black line) of 0.5 M H_2_O_2_. (D) Chopped LSV curves recorded with a bare (black line) and NiFeO_*x*_-modified (red line) CuWO_4_:80 photoanode under monochromatic irradiation at 420 nm; the light intensity is 9 mW cm^–2^.

The PEC characterization of CuWO_4_ photoanodes points to a photoactivity up to 550–560 nm and severe charge carrier recombination due to limited electron mobility in the bulk. To collect further information about this weak point of CuWO_4_ photoefficiency, we sought to investigate the oxidation reaction and stability of this semiconductor. The results of such an analysis are reported in Figure 5.

We first compared the performance of CuWO_4_ to that of an oxide affected by intrinsic sluggish surface water oxidation kinetics. We chose bismuth vanadate because we extensively studied it in previous studies and for its relatively poor photocatalytic activity in the oxygen evolution reaction.^54^ As shown in Figure 5A, the photocurrent response of undoped BiVO_4_ at 1.23 V_SHE_ rapidly decreases by more than 60% within a few seconds after the beginning of irradiation. This behavior is usually ascribed to surface hole accumulation due to the slow hole transfer across the semiconductor/electrolyte interface and consequent charge carrier recombination.

Moreover, when the light is switched off, a negative capacitive current appears for BiVO_4_ due to the consumption of accumulated surface holes via recombination with electrons from the external circuit (a negative current indicates a reductive process taking place at the working electrode). On the other hand, the CuWO_4_ photoanode shows a sharp photocurrent onset and negligible photocurrent decrease under irradiation, and a sharp decrease when the light is turned on and off, respectively (Figure 5A). These behaviors point to low hole accumulation at the CuWO_4_ surface; that is, consumption of surface holes occurs right after they reach the electrode/electrolyte interface. We also checked the stability of the CuWO_4_ electrode and found a negligible activity drop during a 2 h test (see Figure 5B), supporting the low hole accumulation at the surface and the chemical stability of this semiconductor.

To probe the nature of the electron–hole recombination, we performed PEC experiments in the presence of a more feasible oxidation reaction. To do so, we tested the CuWO_4_:580 electrode with an electrolyte containing H_2_O_2_ as an electron donor. Hydrogen peroxide is much more easily oxidizable than water (+0.68 V_SHE_ vs 1.23 V_SHE_ for water) and has a higher reaction rate (H_2_O_2_ to H_2_O is a 2-hole process, while H_2_O to O_2_ is a 4-hole process).^24^ The current doubled to 0.67 mA cm^–2^ at 1.23 V_SHE_ in the presence of H_2_O_2_ (Figure 5C). However, this increase is quite limited compared to BiVO_4_, for which photocurrent increases from a few μA to mA in contact with electron-donor-containing solutions.^55,56^ The moderate photocurrent increase in CuWO_4_ suggests that the few holes reaching the photocatalyst surface can very efficiently undergo oxidation reactions (i.e., water or electron donor oxidation). This experimental evidence rules out sluggish surface electron transfer as the main performance bottleneck in these photoactive films and indicates that the low PEC efficiency of CuWO_4_ stems far from the surface catalytic sites.

To further check this hypothesis, we deposited a nickel–iron oxyhydroxide water oxidation catalyst on top of CuWO_4_. NiFeO_*x*_ ad-layers usually enhance the PEC performance of semiconductors which are limited by poor water oxidation kinetics (e.g., BiVO_4_ or Ta_3_O_5_) or by low chemical stability (Si, or CdTe).^54,57,58^ PEC analyses showed no performance enhancement for the NiFeO_*x*_-modified CuWO_4_ photoanode compared to the bare one (Figure 5D). The negligible effect of the NiFeO_*x*_ cocatalyst confirms the intrinsic activity of surface CuWO_4_ sites and that, therefore, photogenerated charge carriers recombine far from the semiconductor/electrolyte interface.

## 4 Discussion

The spin coating preparation technique employed here offers a simple way to tune the CuWO_4_ thickness and reach a desirable trade-off between visible light absorption and electron–hole separation, resulting in optimal PEC performances. The highest current density achieved (0.37 mA cm^–2^ with the 580 nm thick CuWO_4_ electrode) aligns with the benchmark literature reports (Table 2). The thickness of the best-performing photoanode (580 nm) is similar to that of transparent electrodes prepared by Tian et al. with a spin-coating synthesis (500 nm),^48^ suggesting that the optimal balance between absorption and charge separation in undoped, bulk, and polycrystalline CuWO_4_ is within 5–600 nm. Recently, facet control along the 100 crystal facet showed improved conductivity and enhanced current density (0.38 mA cm^–2^) in bulk 1.5 μm thick CuWO_4_ films.^43^

Nanostructuring provides efficient charge carrier transport.^59^ Indeed, CuWO_4_ electrodes with nanoflake morphology^22,29,44−47,52^ facilitate hole extraction from the oxide flakes because their 30–60 nm width is within the hole diffusion length in CuWO_4_. However, the film thickness (≥1 μm) exceeds electron diffusion in CuWO_4_ and likely limits the PEC performance to ∼0.4 mA cm^–2^.

Doping and mild reduction via hydrogenation of semiconductors offer further control of the defectivity and charge carrier density. For example, Mo doping in BiVO_4_ improves PEC performances by increasing electron mobility, disfavoring recombination, and enhancing charge separation.^60,61^ These strategies induce similar chemical and electronic effects in CuWO_4_ and translate into efficiency improvements (from 0.30 to 0.39 mA cm^–2^ for hydrogenation,^44^ from 0.35 to 0.62 mA cm^–2^ for Mo doping,^22^ from 0.27 to 0.42 mA cm^–2^ for Fe doping,^49,50^ from 0.38 to 0.57 mA cm^–2^ for fluorine doping).^52^

The moderate photocurrent increase observed with CuWO_4_ in the presence of hole scavengers such as H_2_O_2_ (Figure 5C) indicates that few holes reach the electrode/electrolyte interface, where they are rapidly consumed at surface catalytic sites.^41^ This could be behind the slight photocurrent increase reported in previous work with Co-based oxygen evolution cocatalysts (from 0.34 to 0.42 mA cm^–2^ with CoPi,^45^ from 0.32 to 0.54 with CoIrO_*x*_,^47^ and from 0.4 to 0.5 mA cm^–2^ with Co_3_O_4_).^48^ In this work, we observed that the PEC performance of CuWO_4_ photoanodes is unmodified after coating them with NiFeO_*x*_. The lack of change in photoactivity could rely on the ineffective junction between the two materials. These findings further support the fact that Co-based oxygen evolution catalysts are more suitable to enhance the activity of CuWO_4_ than Ni- and Fe-based ones. The simple synthesis reported here could offer a good platform to further enhance the CuWO_4_ performance via these synthetic and postsynthetic approaches.

## 5 Conclusions

CuWO_4_ photoanodes are good candidates for PEC water splitting applications. In fact, CuWO_4_ is very photostable and, compared with other semiconductor oxides, has a high intrinsic activity of the surface catalytic sites for water oxidation. On the other hand, PEC tests under front- and back-side irradiation confirm the low mobility of photoelectrons in CuWO_4_ and indicate that more than 90% of photogenerated charge carriers recombine in the bulk, which heavily limits the photon to current efficiency of CuWO_4_. The higher quantum efficiencies at low light intensities (e.g., IPCE or below 1 sun) indicate that charge separation becomes less efficient at high photogenerated charge carrier densities and that charge recombination occurs mainly in bulk at defects or interface states. Therefore, CuWO_4_ electrode optimization requires reducing this internal charge recombination through: (i) nanostructuring, to facilitate electron extraction toward FTO; (ii) defect engineering, to minimize the presence of recombination centers; and (iii) doping the crystalline structure, to act on the electronic states of the material and modulate its charge transport properties.

## Supporting Information Available

Field emission electron microscopy images, absorbance vs thickness plot, IPCE and IQE plot for the CuWO_4_-350 electrode, IPCE at 1.73 V_SHE_ (PDF)
