# Supplementary material for: Nature of Charge Carrier Recombination in CuWO4 Photoanodes for Photoelectrochemical Water Splitting
Source: ACS Appl Energy Mater. 2023 Sep 20;6(19):10020–9. doi: 10.1021/acsaem.3c01608 (PMC10565723; doi:10.1021/acsaem.3c01608)
Supplement: Supplementary file 1 — ae3c01608_si_001.pdf [file ae3c01608_si_001.pdf]

Supporting information

Nature of Charge Carrier Recombination in $CuWO_4$ Photoanodes for Photoelectrochemical Water Splitting

Ivan Grigioni, Annalisa Polo, Chiara Nomellini, Laura Vigni, Alessandro Poma, Maria Vittoria Dozzi, and Elena Selli*

Dipartimento di Chimica, Università degli Studi di Milano, Via Golgi 19, 20133 Milano, Italy

* Corresponding author, e-mail: ivan.grigioni@unimi.it

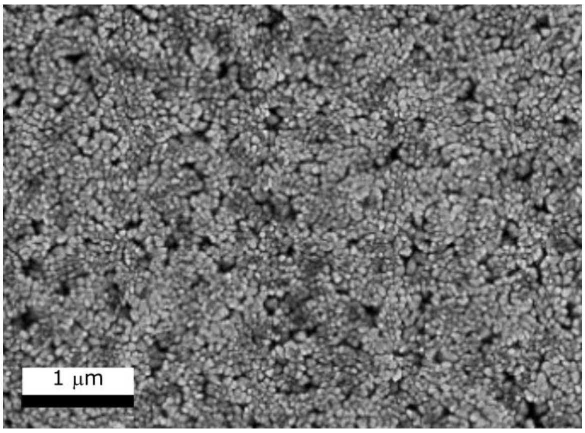

Figure S1. FESEM top view of a $CuWO_4$ electrode obtained after deposition of one layer.

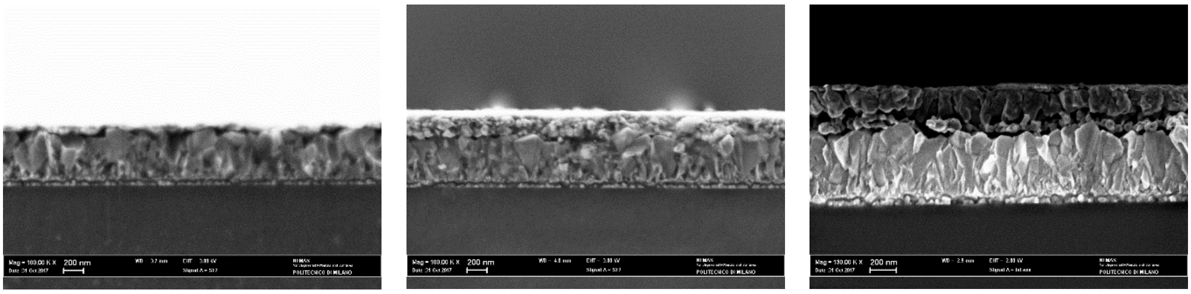

Figure S2. FESEM side view images of $CuWO_4$ electrodes obtained after deposition of A) 1 layer; B) 2 layers; C) 3 layers.

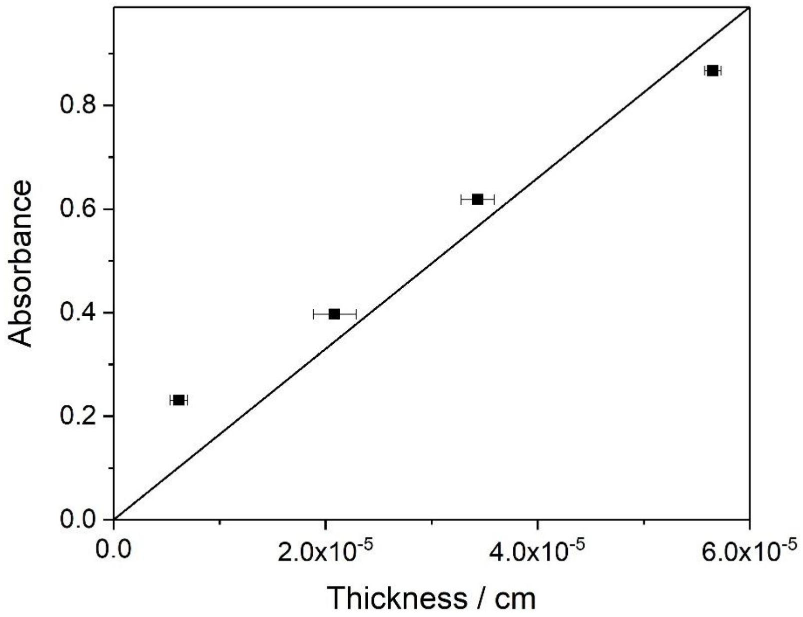

Figure S3. Plot of the absorbance at 420 nm of the $CuWO_4$ films prepared by one, two, three, and five subsequent layer depositions vs. the film thickness.

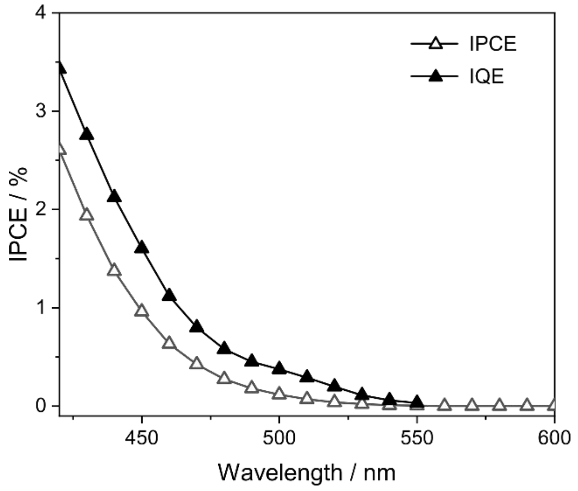

Figure S4. Comparison of the IQE and IPCE curves in the visible region for the $CuWO_4$:350 electrode.

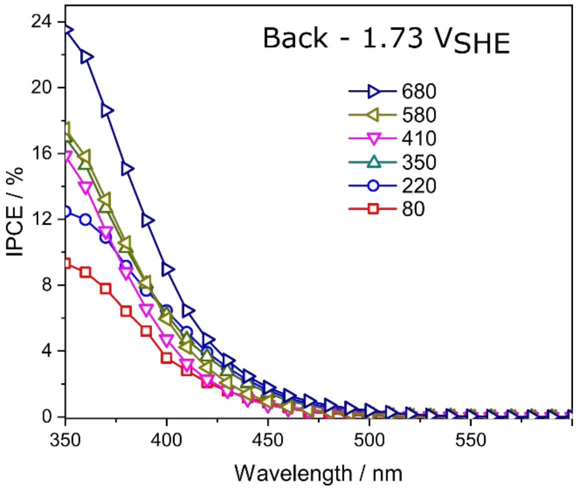

Figure S5. IPCE recorded with the differently thick $CuWO_4$ photoanodes under back-side irradiation at 1.73 V_{SHE}.

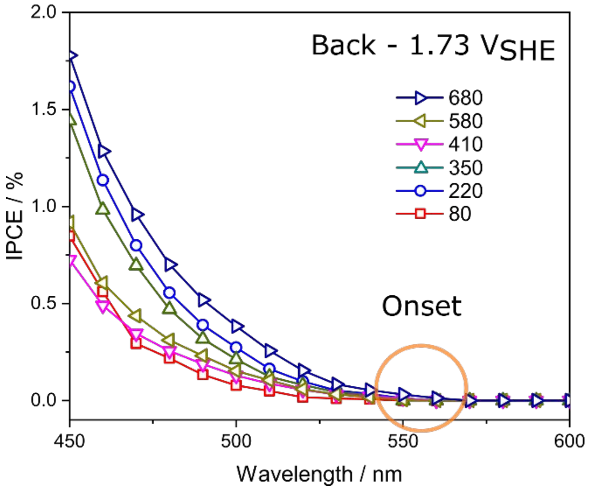

Figure S6. Visible portion of the IPCE curve recorded at 1.73 V_{SHE} with the differently thick $CuWO_4$ photoanodes under back-side irradiation evidencing the onset of the $CuWO_4$ photocurrent response.
